# Liver‐specific changes in *Ppp1r3b* expression impact cognitive function and neuroinflammation in mice

**DOI:** 10.1002/alz.095552

**Published:** 2025-01-09

**Authors:** Varnitha Punnuru, Jacqueline Wu, Amrith Rodrigues, Aisha Wilson, Malaika Mahmood, Emily Lo, Daniel J. Rader, Gregory Corder, Kate Townsend Creasy

**Affiliations:** ^1^ Perelman School of Medicine, University of Pennsylvania, Philadelphia, PA USA; ^2^ School of Arts and Sciences, University of Pennsylvania, Philadelphia, PA USA

## Abstract

**Background:**

Cardiometabolic disorders represent a pressing public health challenge and confer heightened susceptibility to age‐related cognitive decline, including increased risk of developing Alzheimer’s disease (AD). There is a strong genetic component underlying risks for these conditions, and human Genome‐Wide Association Studies (GWAS) have associated the *PPP1R3B* genetic locus with both cardiometabolic dysfunction and AD. PPP1R3B protein is primarily expressed in liver hepatocytes, regulating liver glycogen metabolism. However, there is no clear understanding of its genetic association with AD or its role in the brain. We aim to investigate the molecular mechanisms underlying the association of *PPP1R3B* with AD and to clarify how liver metabolic dysfunction contributes to neurological symptoms.

**Method:**

Hepatocyte‐specific *Ppp1r3b* deletion (KO), over‐expression (OE), and wild‐type (WT) control mice underwent baseline learning and memory assessments by Y‐maze and Novel Object Recognition, and blood samples were collected. Mice were aged to 1 year then underwent additional cognitive assessments and were subsequently euthanized. Brain, liver, and blood samples were collected for assessments of metabolic and neurological dysfunction.

**Result:**

Hepatocyte‐specific *Ppp1r3b* KO mice displayed impaired spatial memory recall as early as four months. Mice with hepatocyte *Ppp1r3b* OE showed no differences in memory compared to WT mice. Brain section immunohistochemistry revealed KO mice exhibited increased activation of microglia in memory‐related brain regions compared to both OE and WT mice. WT mice had some activated microglia while OE mice had less, suggesting OE protection from age‐related microglia dysfunction. Lipidomics performed on brain, liver, and blood samples revealed significant changes in lipid species that suggest a connection between liver metabolic dysfunction and observed cognitive phenotypes.

**Conclusion:**

Hepatocyte‐specific *Ppp1r3b* KO mice develop glucose intolerance and liver metabolic dysfunction that coincides with impaired memory and learning behaviors. After a year of aging, brains from KO mice have increased microglia activation, and altered circulating, brain, and liver lipid profiles that may contribute to the cognitive dysfunction. These data support the role of a liver‐brain axis connecting liver metabolic disease and cognitive impairment. Future studies aim to further clarify the role of specific lipids involved in cardiometabolic disease and AD.

